# Cytolytic T-cell response against Epstein-Barr virus in lung cancer patients and healthy subjects

**DOI:** 10.1186/1756-9966-29-64

**Published:** 2010-06-04

**Authors:** Vaios Karanikas, Maria Zamanakou, Faye Soukou, Theodora Kerenidi, Ioannis Tsougos, Kiki Theodorou, Panagiotis Georgoulias, Konstantinos I Gourgoulianis, Anastasios E Germenis

**Affiliations:** 1 Cancer Immunology Unit, Department of Immunology & Histocompatibility, School of Medicine, University of Thessaly, University Hospital of Larissa, GR-411 10 Larissa, Greece; 2 Department of Respiratory Medicine, School of Medicine, University of Thessaly, University Hospital of Larissa, GR-411 10 Larissa, Greece; 3 Department of Medical Physics, School of Medicine, University of Thessaly, University Hospital of Larissa, GR-411 10 Larissa, Greece; 4 Department of Nuclear Medicine, School of Medicine, University of Thessaly, University Hospital of Larissa, GR-411 10 Larissa, Greece

## Abstract

**Background:**

This study aimed to examine whether EBV seropositive patients with lung cancer have an altered virus-specific CTL response, as compared to age-matched healthy controls and whether any variation in this response could be attributed to senescence.

**Methods:**

Peripheral blood mononuclear cells from lung cancer patients, age-matched and younger healthy individuals were used to measure EBV-specific CTLs after in vitro amplification with the GLCTLVAML and RYSIFFDYM peptides followed by HLA-multimer staining.

**Results:**

Lung cancer patients and aged-matched controls had significantly lesser EBV-specific CTL than younger healthy individuals. Multimer positive populations from either group did not differ with respect to the percentage of multimer positive CTLs and the intensity of multimer binding.

**Conclusions:**

This study provides evidence that patients with lung cancer exhibit an EBV-specific CTL response equivalent to that of age-matched healthy counterparts. These data warrant the examination of whether young individuals have a more robust anti-tumor response, as is the case with the anti-EBV response.

## Introduction

Evidence suggests that cancer patients present with a compromised immune response of multifactorial origin, including the tumor itself. It seems that the early stages of tumor growth appear not to elicit systemic immune deficiency and are sometimes associated with antigen-specific tolerance, while generalized immunodeficiency can arise during the late stages of tumor development [1]. Related data are mainly derived either from in vitro experiments or from DTH measurements in the context of cancer immunotherapy [2]. Therefore, the existing evidence remains inconclusive, while the significance of the described immune alterations in relation to the ability of cancer patients to mount effective responses against pathogens has not been clarified. Finally, there is existing controversy regarding the efficacy of influenza vaccination in patients with cancer [3,4].

This study was scheduled in order to examine whether, at diagnosis, EBV seropositive patients with lung cancer, have a compromised virus-specific CTL response, as compared to age-matched healthy controls. A group of younger healthy individuals was also examined to ascertain whether a possible reduction in the anti-EBV CTL responses of the above patients and age-matched controls could be attributed to senescence. Lung cancer was selected because although such cancers express several tumour antigens [5] and T cells infiltrating these tumours have been identified [6], the outcomes of specific immunotherapy for patients with lung cancer is rather poor [7].

## Subjects and methods

### Patients and controls

PBMC were isolated from whole blood collected at diagnosis from 19 patients with primary lung cancer. Thirteen of them were diagnosed with NSCLC (mean age 66.8 ± 11.8 years; 3 females, 10 males) and the remaining 6 with SCLC (mean age 67.0 ± 7.4 years; 1 female, 5 males). PBMC were also collected from 14 age-matched healthy individuals (mean age 58.2 ± 5.8 years; 4 females, 10 males) as well as from 7 healthy younger individuals (mean age 26.7 ± 1.0 years; 4 females, 3 males). All PBMC were kept frozen till required. Subjects expressed HLA-A2 and/or -A24 (patients: 11 HLA-A2, 6 HLA-A24, 2 HLA-A2/-A24; age-matched healthy individuals: 8 HLA-A2, 5 HLA-A24, 1 HLA-A2/-A24; young healthy individuals: 5 HLA-A2, 2 HLA-A2/-A24) and there were positive for IgG antibodies against the EBV nuclear antigen 3C (EBNA3C). The study conforms to the provisions of the Declaration of Helsinki, it was reviewed and approved by the University of Thessaly Ethics Committee, and all participants provided informed consent.

### Detection of EBV-specific CTLs

Peptide-specific CTLs were detected using HLA-multimer flow cytometry after a previous step of in vitro amplification of MLPCs with peptides under limiting dilution conditions, exactly as described in detail previously [8]. Two EBV peptides, GLCTLVAML (BMLF1.A2 presented by HLA-A2) and RYSIFFDYM (EBNA3C.A24 presented by HLA-A24) were used. These were synthesized on solid phase using F-moc for transient NH2-terminal protection, purchased as lyophilised at > 90% purity ascertained by mass spectrometry (Abgent, San Diego, USA), dissolved in DMSO at 10 mg/mL, and stored at -20 °C before use. Specific multimers labelled with APC and control multimers with PE were used to stain MLPC. Each MLPC was considered to contain a multimer positive population, only if staining with the specific HLA-multimer resulted in a distinct cell cluster that did not stain with control HLA-multimers of different specificity.

### Statistical analysis

Results are expressed as mean ± SD and were analyzed using two tailed chi-square analysis without Yate's correction. The level of significance was 0.05 (two-sided). The commercially available statistical software (SPSS for Windows, release 14.0; SPSS Inc., Chicago, IL.) was used.

## Results

**Figure 1:**
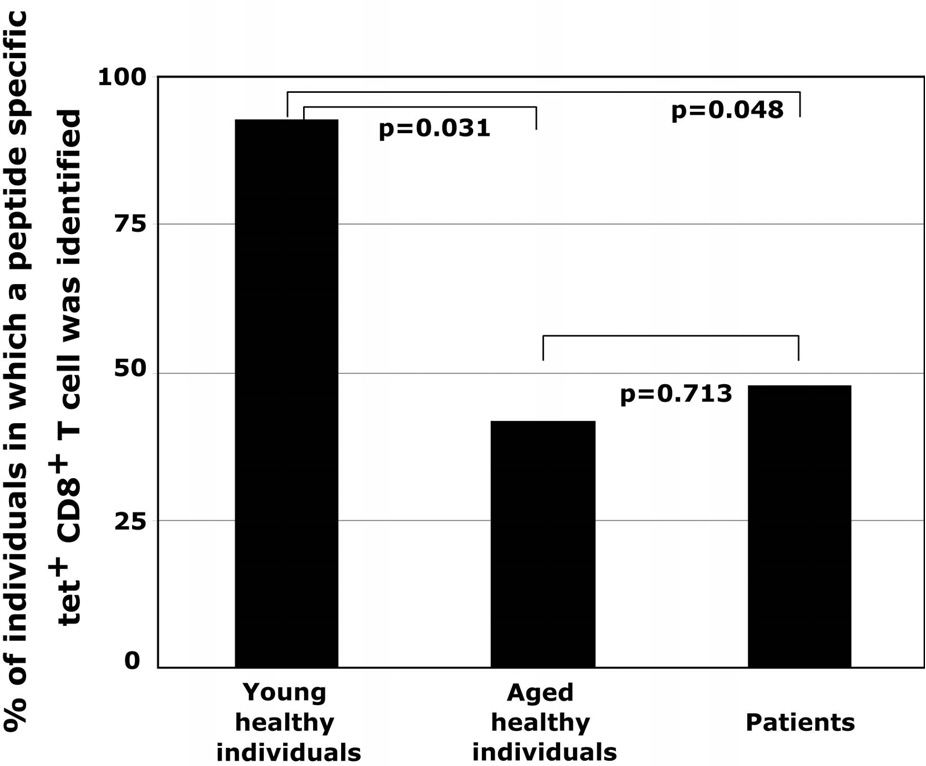
**Proportion of individuals (young healthy, aged healthy and patients) containing an EBV peptide specific tet + CD8 + T cell amongst peripheral blood CD8 T cells**.

EBV-specific CTL responses were detected in the peripheral blood of 8/19 lung cancer patients (42%) and 5/14 (36%) aged-matched controls (p = 0.713). Both of these proportions were statistically significantly different than 86% (6/7) of younger healthy individuals (p = 0.048 and p = 0.031, respectively) that presented with an EBV-specific CTL response (Figure 1). When we examined whether corresponding alterations could be observed against other viruses such as CMV, our findings indicated that the anti-CMV response was similar to that described in the literature [9]. Hence, although all subjects had prior exposure to CMV as determined by serology, younger individuals appeared to have a lesser response as compared to aged individuals (p = 0.046) and aged individuals had a higher response than that observed with patients (p = 0.025) (Table 1).

**Table 1:** Anti-CMV serological response amongst each group

Subject group	**Mean ± Standard deviation**^**a**^	**Range**^**a**^	p
Young healthy individuals	267 ± 183	8-486	Young vs Aged: 0.049
Aged healthy individuals	377 ± 83	411-612	Young vs Patients: 0.466
Patients with lung cancer	341 ± 199	22-831	Aged vs Patients: 0.024

^a^Values represent U/mL of anti-CMV IgG antibodies

Despite the above, the frequency of EBV specific CTL amongst peripheral CD8 T cells was almost the same in the subjects of all groups (Table 2). In all groups, the response against the BMLF1.A2 peptide was more frequent than that against the EBNA3C.A24 peptide (7 patients out of the possible 13, 3 aged-matched controls out of the possible 9 and 6 younger healthy individuals out of the possible 7).

**Table 2:** Number of EBV specific CTL amongst each group

Subject group	**Mean ± Standard deviation**^**a**^	**Range**^**a**^
Young healthy individuals	24.3 ± 17.9	3.1 - 54.8
Aged healthy individuals	25.2 ± 17.2	10.4 - 53.9
Patients with lung cancer	21.8 ± 18.7	1.9 - 60.2

^a^Values represent number of EBV specific CTL amongst one million peripheral CD8 T cells.

**Figure 2:**
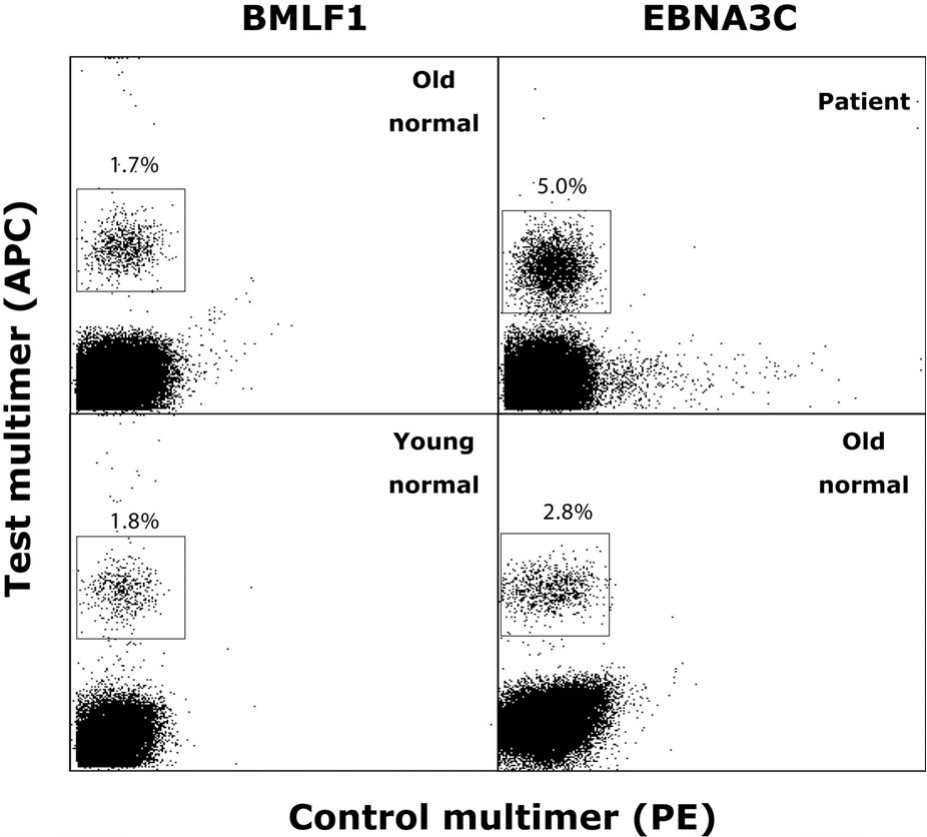
**EBV multimer positive populations from patients, age-matched healthy individuals and healthy younger individuals**. MLPC, were stained with test multimers folded with BMLF1.A2 or EBNA3C.A24 labelled with APC (y axis) and control multimers folded with irrelevant HLA-A2 or -A24 peptides labelled with PE (x axis). Each plot represents live CD8 lymphocytes with the multimer positive population indicated in each gate.

**Figure 3:**
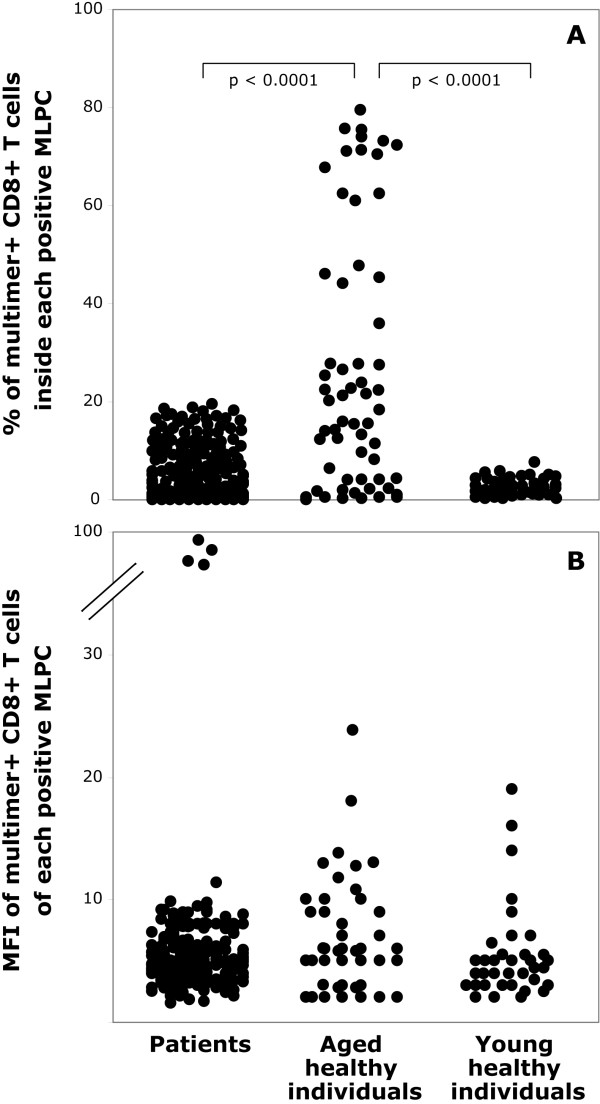
**Flow cytometric characteristics of circulating anti-EBV specific pCTL from patients, age-matched healthy individuals and healthy younger individuals**. *(A)*Percentage of multimer^+^CD8^+ ^T cells inside each positive MLPCs (significance is indicate for comparisons between each group). *(B)*Mean Fluorescence Index (MFI) of HLA-multimers inside the positive MLPCs for each group.

In the process of determining the pCTL frequencies in the peripheral blood, we collected and evaluated flow cytometric data obtained from the analysis of each individual MLPCs. Interestingly, although MLPC containing a multimer positive population, amongst all three groups appeared to have similar multimer positive populations (Figure 2), interesting findings were observed when these were analysed in detail. In particular, the mean percentage of multimer^+^CD8^+ ^T cells inside the positive MLPCs was found significantly higher (p < 0.0001) in age-matched healthy subjects (26.6 ± 26.4%, range 0.4--80.7%) than in lung cancer patients (2.7 ± 3.3%, range 0.1-19.0%) and younger healthy individuals (2.4 ± 1.7%, range 0.2-7.0%) (Figure 3A). This reflects an increased proliferative capacity against the antigenic stimulus of the peptide-specific pCTLs in the older healthy subjects. On the other hand, no statistically significant difference was observed among the three groups with respect to the intensity of multimer binding by each multimer positive population (patients; MFI 6.9 ± 12.3, range 2-115, older healthy subjects; MFI 6.0 ± 4.1, range 2-23, younger healthy subjects; MFI 5.1 ± 3.7, range 2-19) (Figure 3B). This indicates that all antiviral T cells had TCR with a similar avidity towards the peptide/MHC complex and no difference in the kinetics of interaction between TcR and multimer complexes could be observed [10]. Regarding the above, a significant correlation was observed between the percentage of multimer^+^CD8^+ ^and the multimer MFI within the patient (r = 0.15, p < 0.0001) and the aged-matched healthy individual group (r = 0.504, p < 0.0001) but not within the young healthy individual group (r = 0.016, p = 0.435).

Finally, we examined whether the presence of an anti-EBV CTL response lung cancer patients correlated with any clinicopathological parameter (age, sex, performance status, loss of weight, stage of disease etc). No significant correlations were uncovered with either group (Table 3).

**Table 3:** Correlations of anti-EBV T cell response upon diagnosis with clinicopathological parameters

		**Anti-EBV T cell response**^**a**^		
		Yes	No	**p-value**^**b**^
**Age**^**c**^	≤ 65	4 (54; 48-63)	2 (43; 43-59)	0.294
	> 65	4 (74; 69-79)	9 (71; 66-81)	0.515
**Histiotype**	NSCLC	5	8	0.837
	SCLC	3	3	0.734
**Sex**	M	5	10	0.601
	F	3	1	0.231
**Performance Status**^**d**^	0	6	10	0.782
	1	2	1	0.427
**Loss of weight**	< 5%	6	8	0.966
	≥ 5%	2	3	0.932
**Stage**	I-II	5	5	0.684
	III-IV	3	6	0.657
**Survival status**	Alive	5	6	0.657
	Dead	3	5	0.824
**Survival**	Days	843.88 ± 235.59	757.89 ± 292.30	0.512

^a ^Patients were grouped according to whether they had a detectable anti-EBV T cell response; ^b ^p values were obtained after comparing for each group every parameter; ^c ^In parentheses, the median and range is indicated (years); ^d ^ECOG Performance status

## Discussion

This study provides direct evidence that lung cancer patients dispose an EBV-specific CTL response equivalent to that of age-matched healthy counterparts. Moreover, it was demonstrated that the EBV-specific CTL response mounted by subjects of this age group, either with cancer or not, was twice as less than that elicited by younger healthy individuals. Regarding the healthy individuals, our results are in accordance to those reported recently by Colonna-Romano et al [11] demonstrating an inverse correlation between age and the percentage of circulating EBV-specific CTLs. Most likely, these observations can be explained in the context of the complex process of T cell immunosenescence [9,12].

With respect to cancer patients, it is interesting that they present with the same age-related alteration of EBV-specific CTL response as their healthy counterparts. In other words, neither the antigenic burden of the tumor nor any other cancer-related factor affected their ability to mount a CTL response against the virus. Assuming that the CTL response of cancer patients against other pathogens follows a similar pattern of alterations, no special vaccination strategy [4] is required other than that followed for elderly people in general, except when they are under the influence of immunosuppressive therapies. To this end, it must be noted that considering the low frequencies detected in our study population (3-60/million CD8), one has no other alternative but to attempt to amplify these cells first in order to understand their reactivity. This is not unusual since other we and others have confirmed that in most cases CTL responses detected after in vitro stimulation reflect the true number of these cells circulating in vivo [13,14].

Beyond differences observed in the specific pCTL frequency related to age, cancer patients also appeared with a decreased proliferative capacity of virus specific pCTL. Most likely these differences could be explained by replicative senescence [15,16], whereby viral specific CTL in patients have multiplied several times over their lifetime and present with a reduced ability to further respond to an antigenic stimulus. This does not exclude their presence but rather supports the fact that T cell clonal exhaustion results in the accumulation of oligoclonal dysfunctional cells followed by repertoire shrinkage due to clonal deletion, maintaining however, the actual number of dysfunctional cells [17], as has recently being demonstrated in patients with renal cell cancer [18].

Many investigators relate the immune dysfunction of cancer patients with both the inefficient anti-tumor response and a reduced efficacy of immunotherapy [19,20]. To this end, we have recently identified that patients with lung cancer present with a tenfold higher number of anti-tumor CTL as compared to the age-matched controls [13]. These results suggest that such patients do not have an immunocompromised CD8 T cell response but the ineffective anti-tumor response, is most likely a reflection of the age-associated changes that take place in individuals [21] impacting on their capacity to respond effectively against the tumor. Under the light of the data presented herein, it is worth examining whether young individuals have a more robust anti-tumor response, as is the case with the anti-EBV response.

## Conclusions

In conclusion, this study provides evidence that lung cancer patients dispose an EBV-specific CTL response equivalent to that of age-matched healthy counterparts. Our study suggests that possibly the poor outcome of cancer immunotherapeutic approaches in lung cancer can be a result of the underlying effects of senescence on the immune system rather than an inefficient anti-tumor response. These data warrant the examination of whether young individuals have a more robust anti-tumor response, as is the case with the anti-EBV response.

## List of Abbreviations

APC: allophycocyanin; CMV: cytomegalovirus; CTL: cytolytic CD8^+ ^T cell; DTH: delayed-type hypersensitivity; EBV: Eptein-Barr virus; MLPCs: mixed lymphocyte-peptide cultures; NSCLC: non-small cell lung carcinoma; PBMC: peripheral blood mononuclear cells; PE: phycoerythrin; SCLC: small cell lung carcinoma;

## Competing interests

The authors declare that they have no competing interests.

## Authors' contributions

VK and AEG conceived and designed the study, analysed and interpreted the data and drafted the manuscript. MZ and FS carried out most of the experiments. TK collected samples. IT, KT and PG assisted with cell culture. KIG assisted with the critical revision of the manuscript.

## Acknowledgements

This work was supported by (a) a European Union - European Social Fund (75%) and the Greek Ministry of Development-GSRT (25%) (ENTER 04EP09) grant and (b) a Marie Curie Incoming International Fellowship within the 6th European Community Framework Programme (FP6 Contract MIF1-CT-2006-021795, IRTALUNG) grant.
